# Paramagnetic relaxivity of delocalized long-lived states of protons in chains of CH_2_ groups

**DOI:** 10.5194/mr-4-47-2023

**Published:** 2023-02-16

**Authors:** Aiky Razanahoera, Anna Sonnefeld, Geoffrey Bodenhausen, Kirill Sheberstov

**Affiliations:** Department of Chemistry, École Normale Supérieure, PSL University, 75005 Paris, France

## Abstract

Long-lived states (LLSs) have lifetimes $T_{\mathrm{LLS}}$ that can be much longer than longitudinal relaxation times $T_{1}$ . In molecules containing several geminal pairs of protons in neighboring CH ${}_{2}$ groups, it has been shown that *delocalized* LLSs can be excited by converting magnetization into imbalances between the populations of singlet and triplet states of each pair. Since the empirical yield of the conversion and reconversion of observable magnetization into LLSs and back is on the order of 10 % if one uses spin-lock induced crossing (SLIC), it would be desirable to boost the sensitivity by dissolution dynamic nuclear polarization (d-DNP). To enhance the magnetization of nuclear spins by d-DNP, the analytes must be mixed with radicals such as 4-hydroxy-2,2,6,6-tetramethylpiperidin-1-oxyl (TEMPOL). After dissolution, these radicals lead to an undesirable paramagnetic relaxation enhancement (PRE) which shortens not only the longitudinal relaxation times $T_{1}$ but also the lifetimes $T_{\mathrm{LLS}}$ of LLSs. It is shown in this work that PRE by TEMPOL is less deleterious for LLSs than for longitudinal magnetization for four different molecules: 2,2-dimethyl-2-silapentane-5-sulfonate (DSS), homotaurine, taurine, and acetylcholine. The relaxivities $r_{\mathrm{LLS}}$ (i.e., the slopes of the relaxation rate constants $R_{\mathrm{LLS}}$ as a function of the radical concentration) are 3 to 5 times smaller than the relaxivities $r_{1}$ of longitudinal magnetization. Partial delocalization of the LLSs across neighboring CH ${}_{2}$ groups may decrease this advantage, but in practice, this effect was observed to be small, for example, when comparing taurine containing two CH ${}_{2}$ groups and homotaurine with three CH ${}_{2}$ groups. Regardless of whether the LLSs are delocalized or not, it is shown that PRE should not be a major problem for experiments combining d-DNP and LLSs, provided the concentration of paramagnetic species after dissolution does not exceed 1 mM, a condition that is readily fulfilled in typical d-DNP experiments. In bullet d-DNP experiments however, it may be necessary to decrease the concentration of TEMPOL or to add ascorbate for chemical reduction.

## 1 Introduction

The lifetime of spin state populations in nuclear magnetic resonance (NMR) is normally limited by longitudinal relaxation. In certain cases, it is possible to access spin states that have extended lifetimes. In coupled pairs of spins with I=1/2 , such long-lived states (LLSs) correspond to population imbalances between singlet and triplet states (Carravetta and Levitt, 2004; Carravetta et al., 2004) that are immune to intra-pair dipole–dipole interactions, which for pairs of protons are normally the dominant cause of longitudinal relaxation. In larger systems, LLSs may involve four, six, or more spins; all these states are weakly affected by dipolar relaxation (Hogben et al., 2011). The relaxation time constants $T_{\mathrm{LLS}}$ can be much longer than typical longitudinal relaxation time constants $T_{1}$ . This feature is particularly useful for protein–ligand studies (Salvi et al., 2012; Buratto et al., 2014b, 2016). Applications of LLSs can be combined with different hyperpolarization methods, such as parahydrogen-based methods (Franzoni et al., 2012) or dissolution dynamic nuclear polarization (d-DNP) (Bornet et al., 2014; Kiryutin et al., 2019). The latter, d-DNP, is the most universal method to achieve high spin polarization, and it has found applications in drug screening (Lee et al., 2012; Buratto et al., 2014a; Kim et al., 2016) and in studies of metabolism by in vivo magnetic resonance imaging (MRI) (Nelson et al., 2013). Before dissolution, the saturation of the electron spin transitions by microwave irradiation of a solid sample near 1 K leads to an enhancement of the nuclear spin polarization by up to 4 orders of magnitude, compared to the thermal polarization at room temperature in the same magnetic field. The sample is then quickly dissolved and transferred to a solution-state NMR spectrometer, where the high-resolution spectrum is observed (Ardenkjær-Larsen et al., 2003). In an alternative approach known as “bullet DNP”, the cold solid sample is ejected from the polarizer and rapidly transferred to the NMR spectrometer where it is dissolved (Kouřil et al., 2019). After dissolution, the unpaired electrons of the dilute paramagnetic agent give rise to undesirable paramagnetic relaxation enhancement (PRE). For most molecules of interest, such as metabolites or potential drugs, proton relaxation is so fast that the level of hyperpolarization suffers during dissolution and transfer, which is one of the reasons why d-DNP is more often used for ${}^{13}$ C or ${}^{15}$ N rather than for protons. Although molecules that are in enriched ${}^{13}$ C and ${}^{15}$ N offer many possibilities for the excitation of LLSs (Feng et al., 2013; Elliott et al., 2019; Sheberstov et al., 2019), there are several drawbacks of using heteronuclei. Labeled compounds are expensive, and ${}^{13}$ C or ${}^{15}$ N observation is much less sensitive compared to ${}^{1}$ H. After converting proton LLSs back into magnetization, only proton signals of interest are observed, while the background is suppressed. LLSs involving pairs of protons often provide good contrast as protons are often directly exposed to the drug–target interface. On the other hand, the relaxation rate constants of LLSs can be enhanced by mechanisms such as dipolar couplings to solvent nuclei, even with low gyromagnetic ratios, and to paramagnetic species (Kharkov et al., 2022).

Recently, it was discovered that LLSs involving geminal pairs of protons can be readily excited in many molecules containing at least two neighboring CH ${}_{2}$ groups (Sonnefeld et al., 2022a, b). Aliphatic chains, which are the focus of this study, are commonly found in potential drugs, so LLSs of CH ${}_{2}$ groups could provide a new tool for drug screening using NMR. Hyphenation of the LLS methodology with d-DNP offers promising perspectives, since at very low spin temperatures on the order of 10 mK that are routinely achieved in d-DNP singlet–triplet imbalances can result from a violation of the high-temperature approximation, so LLSs can be excited without any radio-frequency (RF) irradiation (Tayler et al., 2012; Bornet et al., 2014; Kress et al., 2019). LLSs that involve chemically equivalent proton pairs in CH ${}_{2}$ groups need not be sustained by RF fields nor protected by shuttling to low fields. Therefore, one can transfer samples with hyperpolarized LLSs to an NMR spectrometer for detection without significant loss of polarization. For small molecules, the ratio $T_{\mathrm{LLS}}/T_{1}$ ranges typically from 2 to 6 for LLSs in CH ${}_{2}$ groups in non-degassed samples (Sonnefeld et al., 2022a), although it is possible to achieve a ratio $T_{\mathrm{LLS}}/T_{1}>30$ in some degassed samples containing isolated pairs of protons (Sarkar et al., 2007) or carbon-13 nuclei (Pileio et al., 2012; Stevanato et al., 2015). In this work, we carried out a systematic analysis of relaxivities, i.e., of the dependence of the relaxation rate constants of LLSs and longitudinal magnetization on the concentration of the paramagnetic species 4-hydroxy-2,2,6,6-tetramethylpiperidin-1-oxyl (TEMPOL).

**Figure 1:**
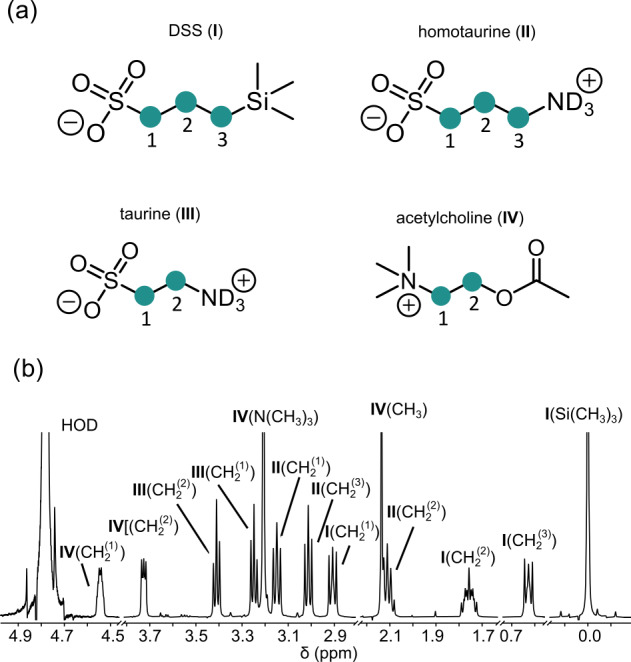
**(a)** Chemical structures of four molecules supporting LLSs of CH ${}_{2}$ groups studied in this work: 2,2-dimethyl-2-silapentane-5-sulfonate sodium salt (DSS, I), homotaurine (II), taurine (III), and acetylcholine (IV). CH ${}_{2}$ groups supporting LLSs are numbered in each structure and highlighted by green circles. **(b)** Assignment of the ${}^{1}$ H NMR spectrum of a mixture containing all four compounds.

Paramagnetic transition metal ions (Cu ${}^{2+}$ , Mn ${}^{2+}$ ), lanthanides (Gd ${}^{3+}$ ), and triplet oxygen (O ${}_{2}$ ) have been shown to induce PRE of LLSs, although PRE is not very efficient as the fluctuating external fields at the sites of two closely spaced protons attached to the same carbon atom are strongly correlated (Tayler and Levitt, 2011). The effects of triplet oxygen on LLSs have been investigated in detail (Erriah and Elliott, 2019). The question arises if fluctuating external fields due to the bulky TEMPOL radical are more strongly correlated than for paramagnetic ions or oxygen, in particular when they act on delocalized LLSs involving several neighboring CH ${}_{2}$ groups in the molecules shown in Fig. 1. In DSS (I) and homotaurine (II), the LLSs can be delocalized over all six protons of the three CH ${}_{2}$ groups, whereas in taurine (III) and acetylcholine (IV) the LLSs always involve all four protons of both CH ${}_{2}$ groups. Titration experiments with TEMPOL allowed us to determine to what extent the radical affects the LLS lifetimes and to determine whether it is necessary to quench the radicals after dissolution (Miéville et al., 2010). In low fields, in particular after dissolution during the transfer between the polarizer and the NMR magnet, PRE may be exacerbated by translational diffusion (Borah and Bryant, 1981) of the paramagnetic molecules relative to the analytes (Miéville et al., 2011).

## 2 Experimental methods

**Figure 2:**
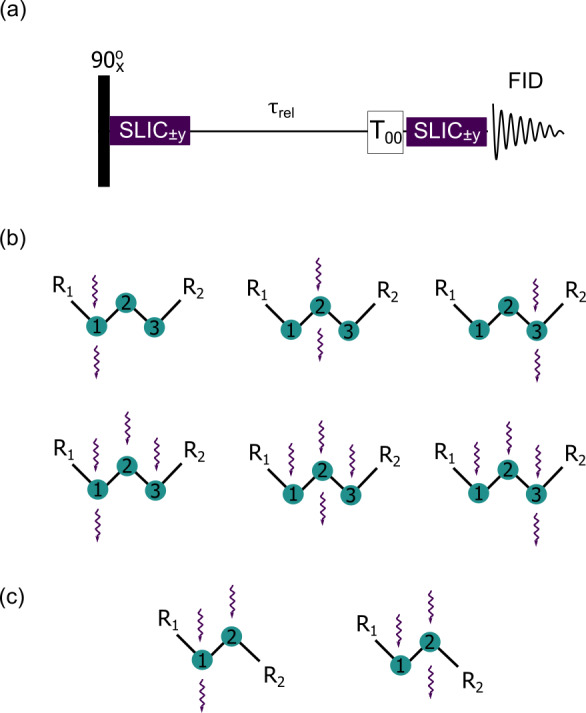
**(a)** Generic pulse sequence for single and poly-SLIC where selective RF fields can be applied simultaneously to two or more CH ${}_{2}$ groups. **(b)** Six possible poly-SLIC experiments applied to molecules containing three CH ${}_{2}$ groups such as I and II of Fig. 1a. The upper row shows three experiments with irradiation at a single frequency for the creation of LLSs and a single readout pulse applied to the offset of the first, second, or third CH ${}_{2}$ group; the lower row shows three experiments using triple irradiation of all three CH ${}_{2}$ groups for LLS excitation, combined with a single readout SLIC applied to only one of the three CH ${}_{2}$ groups. **(c)** Two schemes with double SLIC excitation and single SLIC readout for compounds containing only two CH ${}_{2}$ groups such as III and IV of Fig. 1a.

The delocalized LLSs were excited by using spin-lock induced crossing (SLIC) (DeVience et al., 2013) and its polychromatic extension (Sonnefeld et al., 2022b). A generic SLIC pulse sequence is illustrated in Fig. 2a. After a non-selective 90 ${}^{\circ }$ pulse that rotates the magnetization into the transverse plane, one, two, or three continuous SLIC pulses with a common duration $\tau_{\mathrm{SLIC}}$ are applied to the nuclei of interest, with a common RF amplitude (nutation frequency) $\nu_{1}$ that matches a multiple of the geminal intra-pair J -coupling, i.e., $\nu_{1}=nJ_{\mathrm{HH}}^{\mathrm{intra}}$ with n=1 for double-quantum (DQ) SLIC and n=2 for single-quantum (SQ) SLIC. Level anti-crossings (LACs) lead to a transfer of magnetization into LLSs, i.e., into a population imbalance between states with different permutation symmetry. Since pairs of protons in CH ${}_{2}$ groups are chemically equivalent in achiral molecules (i.e., have the same chemical shifts), and, in the absence of couplings to heteronuclei, are often nearly magnetically equivalent, there is no need to suppress singlet-to-triplet leakage by transporting the sample into a region of low magnetic field nor by applying an RF field to sustain the imbalance. After allowing the LLSs to relax during a delay $\tau_{\mathrm{rel}}$ , a $T_{00}$ filter removes short-lived terms (Tayler and Levitt, 2013; Tayler, 2020), and a second SLIC pulse reconverts the remaining LLSs back into observable magnetization for detection. In this work, SLIC experiments with single, double, and triple irradiation (henceforth called single, double, and triple SLIC for simplicity) were carried out to determine $T_{\mathrm{LLS}}$ , as shown by wavy arrows in Fig. 2b and c.

**Figure 3:**
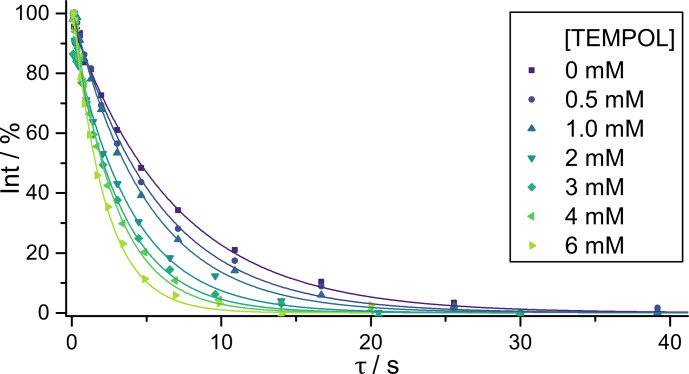
Decays of LLS-derived signals of DSS (compound I) for different TEMPOL concentrations. The LLSs were excited and reconverted by irradiation with single SLIC pulses applied to CH ${}_{2}^{\left(1\right)}$ with an RF amplitude of 27 Hz to match the condition for single-quantum level anti-crossing (SQ LAC). The solid lines correspond to mono-exponential fits, scaled to begin at 100 %.

Titrations were performed by preparing a set of samples where all compounds except TEMPOL had fixed concentrations. The volume of each sample was 600 µ L. A stock solution with 40 mM of each compound was diluted by a factor of 4 to obtain a final concentration of 10 mM for each compound in D ${}_{2}$ O at pH 7.0 without removing paramagnetic oxygen by degassing. A stock solution of phosphate buffer (70 mM KH ${}_{2}$ PO ${}_{4}$ and 130 mM K ${}_{2}$ HPO ${}_{4}$ ) was prepared in D ${}_{2}$ O and diluted by a factor of 4. A 20 mM TEMPOL stock solution was diluted in steps and added to yield final concentrations of 0.5, 1.0, 2.0, 3.0, 4.0, and 6.0 mM. The ${}^{1}$ H NMR spectra were obtained by adding 16 transients (for experiments with single SLIC irradiation) or 8 transients (for experiments with multiple SLIC irradiation) using a 500 MHz AVANCE Neo Bruker spectrometer with a 5 mm iProbe at 298 K. Each sample contained a mixture of all four molecules, thus ensuring accurate comparisons of relaxation rate constants of different molecules. The ${}^{1}$ H NMR spectrum of the mixture with its assignments is presented in Fig. 1b. Typical signal decays due to LLS relaxation as a function of the TEMPOL concentration are shown in Fig. 3. The intensities of the LLS-derived signals are typically about 5 % for single SLIC experiments and up to 10 % for poly-SLIC experiments. The theoretical maximum efficiency of LLS excitation and reconversion in a four-spin –CH ${}_{2}$ –CH ${}_{2}$ – moiety was calculated to be 14 % for single SLIC and 28 % for double SLIC experiments (Sonnefeld et al., 2022a). Simulations of the contributions of different LLS terms to the observed signals were performed using SpinDynamica (Bengs and Levitt, 2018).

## 3 Results and discussion

### 3.1 Comparison of relaxivities of long-lived states and of longitudinal magnetization: partly correlated random fields

**Figure 4:**
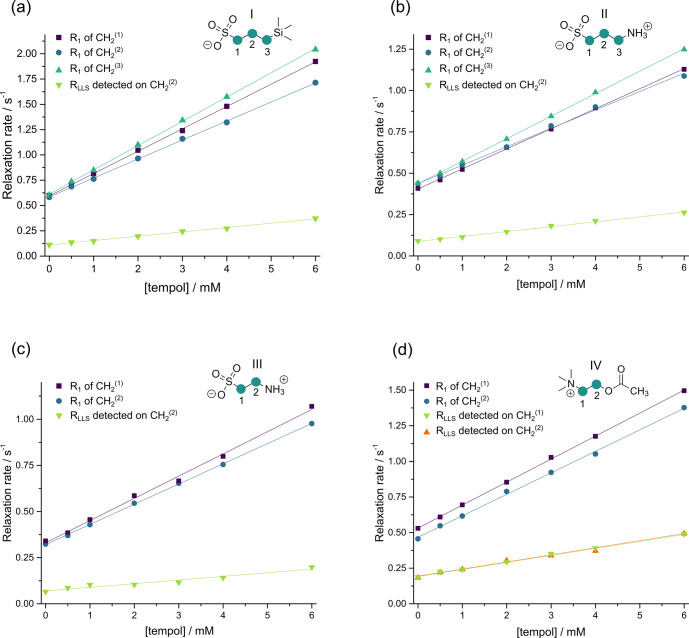
Relaxation rate constants $R_{1}=1/T_{1}$ and $R_{\mathrm{LLS}}=1/T_{\mathrm{LLS}}$ in CH ${}_{2}$ groups of the four molecules I–IV as a function of the TEMPOL concentration. In **(a)** and **(b)**, the LLSs were excited by triple SLIC, in **(c)** and **(d)** by double SLIC, both with an RF amplitude of 13.5 Hz to match the condition for double-quantum level anti-crossing (DQ LAC). In all cases, the LLSs were reconverted into magnetization by single SLIC applied to the CH ${}_{2}^{\left(2\right)}$ group, except for compound IV, where two sets of experiments were performed with reconversion into magnetization of either CH ${}_{2}^{\left(1\right)}$ or CH ${}_{2}^{\left(2\right)}$ groups. The relaxivities $r_{1}$ and $r_{\mathrm{LLS}}$ correspond to the slopes of the linear regressions.

As apparent in Fig. 4, both the longitudinal relaxation rate constant $R_{1}=1/T_{1}$ and the long-lived relaxation rate constant $R_{\mathrm{LLS}}=1/T_{\mathrm{LLS}}$ depend linearly on the concentration of TEMPOL (in units of M or mol L ${}^{-1}$ ):

1 $$\begin{array}{rl} & R_{1}=R_{1}^{\left(0\right)}+r_{1}\;[\mathrm{TEMPOL}],\\ & R_{\mathrm{LLS}}=R_{\mathrm{LLS}}^{\left(0\right)}+r_{\mathrm{LLS}}\;\left[\mathrm{TEMPOL}\right]. \end{array}$$

The slopes $r_{\mathrm{LLS}}$ and $r_{1}$ are known as *relaxivities* (in units of M ${}^{-1}$ s ${}^{-1}$ ); the intercepts $R_{1}^{\left(0\right)}$ and $R_{\mathrm{LLS}}^{\left(0\right)}$ are the rate constants determined in the absence of TEMPOL. Figure 4 shows that variations of $R_{1}$ between neighboring CH ${}_{2}$ groups within each molecule are much smaller than variations from one molecule to another. Whereas the $T_{1}$ values of small molecules correlate with the molecular mass – the larger the molecule, the shorter $T_{1}$ – this is not true for $T_{\mathrm{LLS}}$ . In the absence of TEMPOL, the longest $T_{\mathrm{LLS}}$ of ca. 15 s was observed for compound III, whereas the shortest $T_{\mathrm{LLS}}$ of ca. 5 s was found for compound IV, although their $T_{1}$ relaxation times and molecular masses are roughly the same, so their correlation times should be similar. The difference in $T_{\mathrm{LLS}}$ may be explained by the presence of 12 methyl protons in compound IV, which cause faster relaxation of LLSs.

Wokaun and Ernst famously demonstrated that PRE is less efficient for relaxation of zero-quantum coherences than for single- and double-quantum coherences (Wokaun and Ernst, 1978). Tayler and Levitt demonstrated that a similar logic also applies to LLSs: whereas longitudinal relaxation is enhanced by fluctuations of external local fields induced by unpaired electrons of radicals, a LLS involving two spins $I_{1}$ and $I_{2}$ is only relaxed by fluctuating external fields if these are *not* correlated. In general, the extent of correlation of the two fluctuating fields at the locations of the two spins $I_{1}$ and $I_{2}$ can be characterized by the correlation coefficient $C=\langle B_{1}\cdot B_{2}\rangle /\left(B_{1}B_{2}\right)$ , where $B_{i}=\sqrt{\langle B_{1}\cdot B_{2}\rangle }$ is the mean (time-averaged) amplitude. Only the *uncorrelated* part of the two fluctuating fields given by $\langle B_{1}-B_{2}\rangle^{2}=\left(B_{1}^{2}+B_{2}^{2}-2\langle B_{1}\cdot B_{2}\rangle \right)$ contributes effectively to LLS relaxation (Tayler and Levitt, 2011). The smaller the radical, the closer it can approach one of the two geminal protons and hence the smaller the correlation coefficient C . It has been shown (Tayler and Levitt, 2011) that the ratio of relaxivities,

2 $$\kappa =r_{\mathrm{LLS}}/r_{1},$$

is a characteristic measure of the correlation coefficient C ; the smaller κ , the larger C . The experimental ratio κ for the (chemically inequivalent) protons of the CH ${}_{2}$ group in the (chiral) dipeptide alanine–glycine varied in the range 0.5<κ<0.3 , depending on the size of the paramagnetic agent (Tayler and Levitt, 2011). A similar ratio κ=0.36 was observed for the CH ${}_{2}$ group in the terminal glycine residue of the tripeptide Ala–Gly–Gly for PRE caused by triplet oxygen (Erriah and Elliott, 2019).

In CH ${}_{2}$ chains with chemically equivalent pairs of protons in achiral molecules excited by exploiting magnetic inequivalence, the LLSs can be delocalized over several CH ${}_{2}$ groups. Relaxation of a LLS localized within an individual CH ${}_{2}$ group will contribute to the decay of a delocalized LLS, so one may expect the relaxivity of delocalized LLS to be more strongly affected by PRE than the relaxivity of a (hypothetical) localized LLS. We must however remain cautious, since one cannot assume that all CH ${}_{2}$ groups are equally accessible to radicals. If some of the CH ${}_{2}$ groups are more accessible, one may expect delocalized LLSs to have averaged relaxivities. As we shall discuss below, the variations in the observed relaxivities $r_{\mathrm{LLS}}$ are not very large for different combinations of excitation and reconversion methods, and intramolecular variations are much smaller than differences between distinct compounds, so one can estimate an average ratio of relaxivities $\langle \kappa \rangle =\langle r_{1}\rangle /\langle r_{\mathrm{LLS}}\rangle$ for all CH ${}_{2}$ groups in a given molecule. Compounds I–IV feature average ratios $\langle \kappa_{I}\rangle \approx 0.22$ , $\langle \kappa_{\mathrm{II}}\rangle \approx 0.23$ , $\langle \kappa_{\mathrm{III}}\rangle \approx 0.18$ , and $\langle \kappa_{\mathrm{IV}}\rangle \approx 0.32$ (see Table 1). To assess the effects of LLS delocalization on the relaxivity, it is useful to compare molecules II and III, as they differ by only one CH ${}_{2}$ group. The mean relaxivity $\langle r_{\mathrm{LLS}}\rangle$ of compound II averaged over three CH ${}_{2}$ groups is slightly higher than the relaxivity $\langle r_{\mathrm{LLS}}\rangle$ measured for compound III (see Table 1). This suggests that a higher degree of delocalization leads to a higher relaxivity. The LLS can be delocalized to a variable extent between all three CH ${}_{2}$ groups in I and II, but they are always equally distributed between the two CH ${}_{2}$ groups in compounds III and IV.

**Table 1:** Experimentally determined relaxation rate constants (s ${}^{-1}$ ) and relaxivities (M ${}^{-1}$ s ${}^{-1}$ ). Standard errors determined from linear regressions are shown in parentheses. For double SLIC, the RF amplitude was chosen to match the condition for double-quantum level anti-crossing (LAC), leading to different imbalances characterized by different rate constants $R_{\mathrm{LLS}}^{\left(0\right)}$ (SQ) with single SLIC excitation and single SLIC reconversion, and to rate constants $R_{\mathrm{LLS}}^{\left(0\right)}$ (DQ) with triple SLIC excitation and single SLIC reconversion.

Compound	$R_{1}^{\left(0\right)}$	$R_{\mathrm{LLS}}^{\left(0\right)}$ (SQ)	$R_{\mathrm{LLS}}^{\left(0\right)}$ (DQ)	$r_{1}$	$r_{\mathrm{LLS}}$ (SQ)	$r_{\mathrm{LLS}}$ (DQ)
I, CH ${}_{2}^{\left(1\right)}$	0.596(6)	0.144(2)	0.111(4)	0.221(2)	0.051(1)	0.043(1)
I, CH ${}_{2}^{\left(2\right)}$	0.585(6)	0.116(2)	0.106(3)	0.188(2)	0.046(1)	0.045(1)
I, CH ${}_{2}^{\left(3\right)}$	0.613(5)	0.125(3)	0.113(2)	0.240(2)	0.054(1)	0.048(1)
II, CH ${}_{2}^{\left(1\right)}$	0.405(4)	0.120(2)	0.093(3)	0.121(1)	0.029(1)	0.022(1)
II, CH ${}_{2}^{\left(2\right)}$	0.438(9)	0.102(3)	0.088(3)	0.111(3)	0.032(1)	0.030(1)
II, CH ${}_{2}^{\left(3\right)}$	0.437(3)	0.114(7)	0.089(3)	0.136(1)	0.032(2)	0.022(1)
III, CH ${}_{2}^{\left(1\right)}$	0.33(1)	–	–	0.120(3)	–	–
III, CH ${}_{2}^{\left(2\right)}$	0.321(3)	–	0.069(7)	0.109(1)	–	0.020(2)
IV, CH ${}_{2}^{\left(1\right)}$	0.532(4)	–	0.194(3)	0.162(1)	–	0.050(1)
IV, CH ${}_{2}^{\left(2\right)}$	0.467(8)	–	0.191(6)	0.151(3)	–	0.049(2)

### 3.2 Implications for dissolution DNP

Even though delocalized LLSs are less affected by TEMPOL than longitudinal magnetization, the observed decrease in $T_{\mathrm{LLS}}$ is undesirable in the context of d-DNP. Since the use of TEMPOL or other polarizing agents is mandatory for d-DNP experiments, the question arises if it is worth scavenging TEMPOL after dissolution by addition of a reducing agent such as sodium ascorbate (vitamin C) to extend $T_{\mathrm{LLS}}$ after dissolution (Miéville et al., 2010, 2011). Note that the preparation of samples comprising two types of beads is rather cumbersome, in particular for bullet DNP. According to Miéville et al. (2010, 2011), the rate of the reduction of TEMPOL by sodium ascorbate may be slow on the timescale of the transfer of the dissolved sample from the polarizer to the NMR magnet. Hence, the reaction may not be entirely completed by the time the sample arrives in the spectrometer. Scavenging by sodium ascorbate may be accelerated ca. 100 times if one uses Frémy's salt instead of TEMPOL (Negroni et al., 2022). Several alternative approaches have been developed to remove radicals once DNP has been achieved. One approach is to use radicals obtained by UV irradiation of frozen pyruvic acid. These radicals are quenched as soon as the temperature increases (Eichhorn et al., 2013). One may also use radicals grafted onto mesostructured silica materials (Gajan et al., 2014) or microporous polymers (Ji et al., 2017; El Daraï et al., 2021). However, the small relaxivities presented in Table 1 suggest that scavenging may not be necessary when using LLSs to preserve the hyperpolarization.

### 3.3 Experiments and simulations for molecules with three CH ${}_{2}$ groups

It was shown (Sonnefeld et al., 2022b) that for the excitation of LLSs in systems with n=3 neighboring CH ${}_{2}$ groups, i.e., with 2n=6 spins, there are seven orthogonal LLS product operators that can be created, with seven coefficients $\lambda_{i}$ that depend on the excitation scheme:

3 $$\begin{array}{rl} {\hat{\sigma }}_{\mathrm{LLS}} & =-\lambda_{AA^{\prime }}{\hat{I}}^{A}\cdot {\hat{I}}^{A^{\prime }}-\lambda_{MM^{\prime }}{\hat{I}}^{M}\cdot {\hat{I}}^{M^{\prime }}-\lambda_{XX^{\prime }}{\hat{I}}^{X}\cdot {\hat{I}}^{X^{\prime }}\\ & -\lambda_{AA^{\prime }MM^{\prime }}\left({\hat{I}}^{A}\cdot {\hat{I}}^{A^{\prime }}\right)\left({\hat{I}}^{M}\cdot {\hat{I}}^{M^{\prime }}\right)\\ & -\lambda_{AA^{\prime }XX^{\prime }}\left({\hat{I}}^{A}\cdot {\hat{I}}^{A^{\prime }}\right)\left({\hat{I}}^{X}\cdot {\hat{I}}^{X^{\prime }}\right)\\ & -\lambda_{MM^{\prime }XX^{\prime }}\left({\hat{I}}^{M}\cdot {\hat{I}}^{M^{\prime }}\right)\left({\hat{I}}^{X}\cdot {\hat{I}}^{X^{\prime }}\right)\\ & -\lambda_{AA^{\prime }MM^{\prime }XX^{\prime }}\left({\hat{I}}^{A}\cdot {\hat{I}}^{A^{\prime }}\right)\left({\hat{I}}^{M}\cdot {\hat{I}}^{M^{\prime }}\right)\left({\hat{I}}^{X}\cdot {\hat{I}}^{X^{\prime }}\right). \end{array}$$

Here A and $A^{\prime }$ denote the two protons of the CH ${}_{2}^{\left(1\right)}$ group, M and $M^{\prime }$ denote those of the middle CH ${}_{2}^{\left(2\right)}$ group, and X and $X^{\prime }$ correspond to the terminal CH ${}_{2}^{\left(3\right)}$ group. This equation gives a general form of the density operator obtained after poly-SLIC, containing all long-lived terms found by numerical solution of the Liouville–von-Neumann equation. In addition to three bilinear terms, one encounters four higher terms that contain products of four and six spin operators. In principle, each term in Eq. (3) can decay with a different rate constant, so one could distinguish up to seven distinct rate constants $R_{\mathrm{LLS}}^{\left(\mu \right)}$ with $\mu =AA^{\prime }$ , $MM^{\prime }$ , $XX^{\prime }$ , $AA^{\prime }MM^{\prime }$ , $AA^{\prime }XX^{\prime }$ , $MM^{\prime }XX^{\prime }$ , and $AA^{\prime }MM^{\prime }XX^{\prime }$ . Each term can be excited with a different amplitude and can contribute with a different weight to the observed signal.

In systems such as compounds III and IV with only two CH ${}_{2}$ groups, only one LLS can be excited:

4 $$\begin{array}{rl} {\hat{\sigma }}_{\mathrm{LLS}} & =-\lambda_{AA^{\prime }}{\hat{I}}^{A}\cdot {\hat{I}}^{A^{\prime }}-\lambda_{XX^{\prime }}{\hat{I}}^{X}\cdot {\hat{I}}^{X^{\prime }}\\ & -\lambda_{AA^{\prime }XX^{\prime }}\left({\hat{I}}^{A}\cdot {\hat{I}}^{A^{\prime }}\right)\left({\hat{I}}^{X}\cdot {\hat{I}}^{X^{\prime }}\right). \end{array}$$

The coefficients of the first two bilinear terms are always equal, i.e., $\lambda_{AA^{\prime }}=\lambda_{XX^{\prime }}$ , while the four-spin term is always proportional to the leading bilinear terms, with a weight $\lambda_{AA^{\prime }XX^{\prime }}=8/3\lambda_{AA^{\prime }}$ (Sonnefeld et al., 2022a). This state corresponds to the imbalance between the singlet–singlet state and the triplet–triplet manifold and is therefore expected to decay monoexponentially. In two sets of complementary experiments performed for compound IV, the experimental relaxation rate constants were indeed found to be indistinguishable, as can be seen by comparing the orange triangles and the green inverted triangles in Fig. 4d.

**Figure 5:**
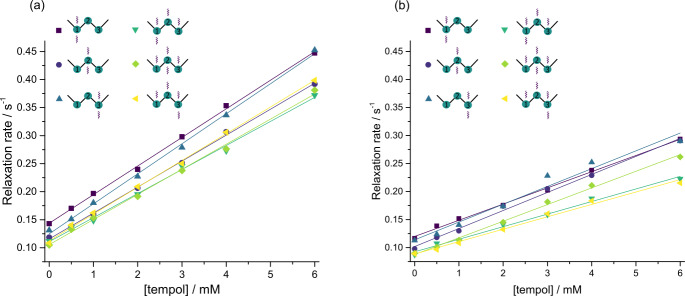
Decay rate constants $R_{\mathrm{LLS}}=1/T_{\mathrm{LLS}}$ of long-lived states in CH ${}_{2}$ groups in **(a)** DSS (I) and **(b)** homotaurine (II), each containing three CH ${}_{2}$ groups, as a function of the TEMPOL concentration. Six different poly-SLIC experiments with distinct excitation and reconversion methods were performed for each molecule, as indicated by wavy arrows. The relaxivities $r_{\mathrm{LLS}}$ correspond to the slopes of the linear regressions.

In compounds I and II however, which contain three adjacent CH ${}_{2}$ groups, different SLIC excitation schemes lead to populate different LLSs, with different coefficients $\lambda_{\mathrm{LLS}}^{\left(\mu \right)}$ in Eq. (3). There are 9 different ways of exciting miscellaneous LLSs and 9 different ways of reconverting them, giving 81 possible experimental combinations. In order to investigate the relaxivities of these different LLSs which may have different decay rate constants $R_{\mathrm{LLS}}^{\left(\mu \right)}$ and different relaxivities $r_{\mathrm{LLS}}^{\left(\mu \right)}$ , we performed six different poly-SLIC experiments with different SLIC pulses for excitation and reconversion, and we indeed found different LLS lifetimes (Fig. 5). Depending on the excitation and reconversion scheme used, there are pronounced differences between the relaxivities $r_{\mathrm{LLS}}$ within one and the same molecule.

**Figure 6:**
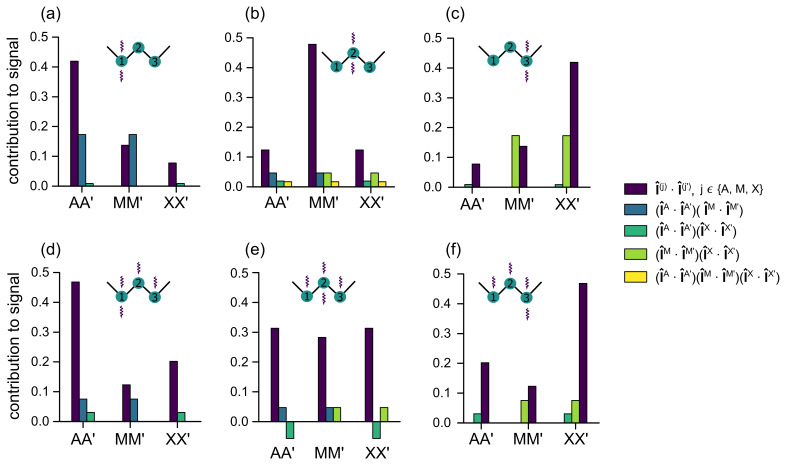
Calculated contributions of the seven different LLS terms ${\hat{P}}_{\mu }$ (the 3 two-spin terms are shown in the same color) in the density operator of Eq. (3) to the observed signals for all six different single and poly-SLIC experiments used in this work to determine the relaxivities $r_{\mathrm{LLS}}^{\left(a\right)}$ in the six-spin systems of DSS (I) and homotaurine (II). The histograms show the products, $\lambda_{\mu }^{M\to \mathrm{LLS}}{\tilde{\lambda }}_{\mu }^{\mathrm{LLS}\to M}$ , of the coefficients of LLS excitation and reconversion methods. The normalization ensures that the sum of all products of coefficients is equal to 1. Experiments with triple SLIC excitation and single SLIC reconversion applied to the middle CH ${}_{2}$ group **(e)** provide LLSs that are almost evenly distributed among all three CH ${}_{2}$ groups, whereas the other experiments provide access to LLSs that are in part localized on the group where the reconversion SLIC pulse is applied. The excitation and reconversion of the (yellow) six-spin term is negligible except for case **(b)**.

We calculated the contributions of each of the seven terms to the observable LLS-derived signals, after two consecutive transformations: ${\hat{I}}_{z}^{\mathrm{in}}\to {\hat{\sigma }}_{\mathrm{LLS}}\to {\hat{I}}_{x}^{\mathrm{obs}}$ (see Fig. 6). For each excitation scheme used in this work, we considered all seven coefficients $\lambda_{\mu }^{M\to \mathrm{LLS}}$ corresponding to the seven terms in Eq. (3), as well as all seven reconversion coefficients ${\tilde{\lambda }}_{\mu }^{\mathrm{LLS}\to M}$ . The coefficients were calculated as follows:

5 $$\begin{array}{rl} & \lambda_{\mu }^{M\to \mathrm{LLS}}({\hat{I}}_{z}^{\mathrm{in}}\to {\hat{\sigma }}_{\mathrm{LLS}})=\frac{\mathrm{Tr}\left\{{\hat{P}}_{\mu }^{†}{\hat{\sigma }}_{\mathrm{LLS}}\right\}}{\mathrm{Tr}\left\{{\hat{P}}_{\mu }^{†}{\hat{P}}_{\mu }\right\}},\\ & {\tilde{\lambda }}_{\mu }^{\mathrm{LLS}\to M}({\hat{P}}_{µ}\to {\hat{I}}_{x,µ}^{\mathrm{obs}})=\frac{\mathrm{Tr}\left\{{\hat{I}}_{x}^{†}{\hat{I}}_{x,\mu }^{\mathrm{obs}}\right\}}{\mathrm{Tr}\left\{{\hat{I}}_{x}^{†}{\hat{I}}_{x}\right\}}, \end{array}$$

where Tr stands for trace and where the index μ corresponds to one of the seven LLS terms in Eq. (3), the operator ${\hat{P}}_{\mu }$ represents the μ th LLS term, ${\hat{I}}_{z}^{\mathrm{in}}$ is the initial magnetization of the excited spins, ${\hat{I}}_{x}$ is the transverse magnetization of the observed spins after reconversion, and ${\hat{I}}_{x,\mu }^{\mathrm{obs}}$ is the transverse magnetization obtained after reconversion of only the μ th term ${\hat{P}}_{\mu }$ instead of the full ${\hat{\sigma }}_{\mathrm{LLS}}$ . The observed signal $S_{\mu }$ stemming from the μ th term is determined by the product of two coefficients, $\lambda_{\mu }^{M\to \mathrm{LLS}}$ and ${\tilde{\lambda }}_{\mu }^{\mathrm{LLS}\to M}$ , for a given combination of excitation and reconversion SLIC pulses. These contributions are shown in Fig. 6. The sum of all seven amplitudes for each panel in Fig. 6 was normalized to one. These graphs show how the LLSs are delocalized across spin systems comprising n=3 neighboring CH ${}_{2}$ groups. We only consider coherent spin dynamics during excitation and reconversion, neglecting possible redistributions of LLSs due to Overhauser-type cross-relaxation effects and neglecting zero-quantum coherences.

Note that a *single* SLIC pulse applied at the chemical shift of *any* of the three CH ${}_{2}$ groups results in the excitation of a delocalized state, which is predominantly (but not exclusively) associated with the irradiated pair. By using triple SLIC excitation and single SLIC reconversion applied to the middle CH ${}_{2}$ group, one can excite a fairly even distribution of LLSs involving all 2n=6 coupled spins. For compound II, the most strongly delocalized state features the largest relaxivity $r_{\mathrm{LLS}}$ . For compound I, however, the largest relaxivities were obtained for experiments where the largest contribution to the observed signal came from the terminal group CH ${}_{2}^{\left(3\right)}$ that is closest to the trimethylsilane group. This group has also the largest longitudinal relaxivity $r_{1}$ , as can be seen in Fig. 4a. Detailed calculations of the relaxation superoperator might help to rationalize the experimental results obtained here.

## 4 Conclusions

The relaxation rate constants of various long-lived states and of the longitudinal magnetization of DSS, homotaurine, taurine, and acetylcholine were measured as a function of the concentration of the radical TEMPOL. In all cases, the relaxivities $r_{\mathrm{LLS}}$ are lower by about a factor of 3 compared to the relaxivities $r_{1}$ . This implies that the effects of paramagnetic relaxation enhancement on LLSs due to TEMPOL during sample transfer in dissolution DNP should not be too severe. Furthermore, the LLS relaxivity was studied for different SLIC excitation and reconversion schemes. The results support simulations that show that different LLSs are excited depending on the SLIC sequence and the number of adjacent methylene units. SLIC methods have also been shown to be efficient for other achiral molecules containing neighboring CH ${}_{2}$ groups, such as dopamine, γ -aminobutyric acid (GABA), ethanolamine, and β -alanine (Sonnefeld et al., 2022a). All of these molecules contain aliphatic chains, so the effects of paramagnetic polarizing agents like TEMPOL should be similar to what is reported in this work.

## Data Availability

All original NMR data obtained for this paper are available through the Zenodo repository under https://doi.org/10.5281/zenodo.7432635 (Razanahoera et al., 2022).

## References

[bib1.bib1] Ardenkjær-Larsen JH, Fridlund B, Gram A, Hansson G, Hansson L, Lerche MH, Servin R, Thaning M, Golman K (2003). Increase in signal-to-noise ratio of 
>
 10,000 times in liquid-state NMR. P Natl Acad Sci USA.

[bib1.bib2] Bengs C, Levitt MH (2018). SpinDynamica: Symbolic and numerical magnetic resonance in a Mathematica environment. Magn Reson Chem.

[bib1.bib3] Borah B, Bryant RG (1981). NMR relaxation dispersion in an aqueous nitroxide system. J Chem Phys.

[bib1.bib4] Bornet A, Ji X, Mammoli D, Vuichoud B, Milani J, Bodenhausen G, Jannin S (2014). Long-Lived States of Magnetically Equivalent Spins Populated by Dissolution-DNP and Revealed by Enzymatic Reactions. Eur J Chem.

[bib1.bib5] Buratto R, Bornet A, Milani J, Mammoli D, Vuichoud B, Salvi N, Singh M, Laguerre A, Passemard S, Gerber-Lemaire S, Jannin S, Bodenhausen G (2014). Drug Screening Boosted by Hyperpolarized Long-Lived States in NMR. ChemMedChem.

[bib1.bib6] Buratto R, Mammoli D, Chiarparin E, Williams G, Bodenhausen G (2014). Exploring Weak Ligand–Protein Interactions by Long-Lived NMR States: Improved Contrast in Fragment-Based Drug Screening. Angew Chem Int Edit.

[bib1.bib7] Buratto R, Mammoli D, Canet E, Bodenhausen G (2016). Ligand–Protein Affinity Studies Using Long-Lived States of Fluorine-19 Nuclei. J Med Chem.

[bib1.bib8] Carravetta M, Levitt MH (2004). Long-Lived Nuclear Spin States in High-Field Solution NMR. J Am Chem Soc.

[bib1.bib9] Carravetta M, Johannessen OG, Levitt MH (2004). Beyond the T1 Limit: Singlet Nuclear Spin States in Low Magnetic Fields. Phys Rev Lett.

[bib1.bib10] DeVience SJ, Walsworth RL, Rosen MS (2013). Preparation of Nuclear Spin Singlet States Using Spin-Lock Induced Crossing. Phys Rev Lett.

[bib1.bib11] Eichhorn T R, Takado Y, Salameh N, Capozzi A, Cheng T, Hyacinthe J-N, Mishkovsky M, Roussel C, Comment A (2013). Hyperpolarization without persistent radicals for in vivo real-time metabolic imaging. P Natl Acad Sci USA.

[bib1.bib12] El Daraï T, Cousin SF, Stern Q, Ceillier M, Kempf J, Eshchenko D, Melzi R, Schnell M, Gremillard L, Bornet A, Milani J, Vuichoud B, Cala O, Montarnal D, Jannin S (2021). Porous functionalized polymers enable generating and transporting hyperpolarized mixtures of metabolites. Nat Commun.

[bib1.bib13] Elliott SJ, Kadeøávek P, Brown LJ, Sabba M, Glöggler S, O'Leary DJ, Brown RCD, Ferrage F, Levitt MH (2019). Field-cycling long-lived-state NMR of 
${}^{15}$
N
${}_{2}$
 spin pairs. Mol Phys.

[bib1.bib14] Erriah B, Elliott SJ (2019). Experimental evidence for the role of paramagnetic oxygen concentration on the decay of long-lived nuclear spin order. RSC Adv.

[bib1.bib15] Feng Y, Theis T, Liang X, Wang Q, Zhou P, Warren WS (2013). Storage of Hydrogen Spin Polarization in Long-Lived 
${}^{13}$
C
${}_{2}$
 Singlet Order and Implications for Hyperpolarized Magnetic Resonance Imaging. J Am Chem Soc.

[bib1.bib16] Franzoni MB, Buljubasich L, Spiess HW, Münnemann K (2012). Long-Lived 
${}^{1}$
H Singlet Spin States Originating from Para-Hydrogen in Cs-Symmetric Molecules Stored for Minutes in High Magnetic Fields. J Am Chem Soc.

[bib1.bib17] Gajan D, Bornet A, Vuichoud B, Milani J, Melzi R, van Kalkeren HA, Veyre L, Thieuleux C, Conley MP, Grüning WR, Schwarzwälder M, Lesage A, Copéret C, Bodenhausen G, Emsley L, Jannin S (2014). Hybrid polarizing solids for pure hyperpolarized liquids through dissolution dynamic nuclear polarization. P Natl Acad Sci USA.

[bib1.bib18] Hogben HJ, Hore PJ, Kuprov I (2011). Multiple decoherence-free states in multi-spin systems. J Magn Reson.

[bib1.bib19] Ji X, Bornet A, Vuichoud B, Milani J, Gajan D, Rossini AJ, Emsley L, Bodenhausen G, Jannin S (2017). Transportable hyperpolarized metabolites. Nat Commun.

[bib1.bib20] Kharkov B, Duan X, Rantaharju J, Sabba M, Levitt MH, Canary JW, Jerschow A (2022). Weak nuclear spin singlet relaxation mechanisms revealed by experiment and computation. Phys Chem Chem Phys.

[bib1.bib21] Kim Y, Liu M, Hilty C (2016). Parallelized Ligand Screening Using Dissolution Dynamic Nuclear Polarization. Anal Chem.

[bib1.bib22] Kiryutin AS, Rodin BA, Yurkovskaya AV, Ivanov KL, Kurzbach D, Jannin S, Guarin D, Abergel D, Bodenhausen G (2019). Transport of hyperpolarized samples in dissolution-DNP experiments. Phys Chem Chem Phys.

[bib1.bib23] Kouøil K, Kouøilová H, Bartram S, Levitt MH, Meier B (2019). Scalable dissolution-dynamic nuclear polarization with rapid transfer of a polarized solid. Nat Commun.

[bib1.bib24] Kress T, Walrant A, Bodenhausen G, Kurzbach D (2019). Long-Lived States in Hyperpolarized Deuterated Methyl Groups Reveal Weak Binding of Small Molecules to Proteins. J Phys Chem Lett.

[bib1.bib25] Lee Y, Zeng H, Ruedisser S, Gossert AD, Hilty C (2012). Nuclear Magnetic Resonance of Hyperpolarized Fluorine for Characterization of Protein–Ligand Interactions. J Am Chem Soc.

[bib1.bib26] Miéville P, Ahuja P, Sarkar R, Jannin S, Vasos PR, Gerber-Lemaire S, Mishkovsky M, Comment A, Gruetter R, Ouari O, Tordo P, Bodenhausen G (2010). Scavenging Free Radicals To Preserve Enhancement and Extend Relaxation Times in NMR using Dynamic Nuclear Polarization. Angew Chem Int Edit.

[bib1.bib27] Miéville P, Jannin S, Bodenhausen G (2011). Relaxometry of insensitive nuclei: Optimizing dissolution dynamic nuclear polarization. J Magn Reson.

[bib1.bib28] Negroni M, Turhan E, Kress T, Ceillier M, Jannin S, Kurzbach D (2022). Frémy's Salt as a Low-Persistence Hyperpolarization Agent: Efficient Dynamic Nuclear Polarization Plus Rapid Radical Scavenging. J Am Chem Soc.

[bib1.bib29] Nelson SJ, Kurhanewicz J, Vigneron DB, Larson PEZ, Harzstark AL, Ferrone M, van Criekinge M, Chang JW, Bok R, Park I, Reed G, Carvajal L, Small EJ, Munster P, Weinberg VK, Ardenkjaer-Larsen JH, Chen AP, Hurd RE, Odegardstuen L-I, Robb FJ, Tropp J, Murray JA (2013). Metabolic Imaging of Patients with Prostate Cancer Using Hyperpolarized [1-13C]Pyruvate. Sci Transl Med.

[bib1.bib30] Pileio G, Hill-Cousins JT, Mitchell S, Kuprov I, Brown LJ, Brown RCD, Levitt MH (2012). Long-Lived Nuclear Singlet Order in Near-Equivalent 
${}^{13}$
C Spin Pairs. J Am Chem Soc.

[bib1.bib31] Razanahoera A, Sonnefeld A, Bodenhausen G, Sheberstov K (2022). Zenodo [data set].

[bib1.bib32] Salvi N, Buratto R, Bornet A, Ulzega S, Rentero Rebollo I, Angelini A, Heinis C, Bodenhausen G (2012). Boosting the Sensitivity of Ligand–Protein Screening by NMR of Long-Lived States. J Am Chem Soc.

[bib1.bib33] Sarkar R, Vasos PR, Bodenhausen G (2007). Singlet-State Exchange NMR Spectroscopy for the Study of Very Slow Dynamic Processes. J Am Chem Soc.

[bib1.bib34] Sheberstov KF, Vieth H-M, Zimmermann H, Rodin BA, Ivanov KL, Kiryutin AS, Yurkovskaya AV (2019). Generating and sustaining long-lived spin states in 
${}^{15}$
N, 
${}^{15}$
N'-azobenzene,. Sci Rep.

[bib1.bib35] Sonnefeld A, Razanahoera A, Pelupessy P, Bodenhausen G, Sheberstov K (2022). Long-lived states of methylene protons in achiral molecules. Sci Adv.

[bib1.bib36] Sonnefeld A, Bodenhausen G, Sheberstov K (2022). Polychromatic Excitation of Delocalized Long-Lived Proton Spin States in Aliphatic Chains. Phys Rev Lett.

[bib1.bib37] Stevanato G., Hill-Cousins JT, Håkansson P, Roy SS, Brown LJ, Brown RCD, Pileio G, Levitt MH (2015). A Nuclear Singlet Lifetime of More than One Hour in Room-Temperature Solution. Angew Chem Int Edit.

[bib1.bib38] Tayler M. C. D., Pileio G (2020). Chapter 10: Filters for Long-lived Spin Order, in: Long-lived Nuclear Spin Order.

[bib1.bib39] Tayler MCD, Levitt MH (2011). Paramagnetic relaxation of nuclear singlet states. Phys Chem Chem Phys.

[bib1.bib40] Tayler MCD, Levitt MH (2013). Accessing Long-Lived Nuclear Spin Order by Isotope-Induced Symmetry Breaking. J Am Chem Soc.

[bib1.bib41] Tayler MCD, Marco-Rius I, Kettunen MI, Brindle KM, Levitt MH, Pileio G (2012). Direct Enhancement of Nuclear Singlet Order by Dynamic Nuclear Polarization. J Am Chem Soc.

[bib1.bib42] Wokaun A, Ernst RR (1978). The use of multiple quantum transitions for relaxation studies in coupled spin systems. Mol Phys.

